# A Combination of *Lactoplantibacillus plantarum* Strains CECT7527, CECT7528, and CECT7529 Plus Monacolin K Reduces Blood Cholesterol: Results from a Randomized, Double-Blind, Placebo-Controlled Study

**DOI:** 10.3390/nu13041206

**Published:** 2021-04-06

**Authors:** Rafael Guerrero-Bonmatty, Guadalupe Gil-Fernández, Francisco José Rodríguez-Velasco, Jordi Espadaler-Mazo

**Affiliations:** 1 Department of Nursing, Merida University Center, University of Extremadura, Av. Santa Teresa de Jornet 38, 06800 Mérida, Spain; rafaelbonmatty@unex.es; 2 Department of Nursing, Faculty of Medicine and Health Sciences, University of Extremadura, Av. Elvas s/n, 06006 Badajoz, Spain; 3 AB-BIOTICS SA, ESADE Creapolis 3B011, Av. Torre Blanca 57, 08172 Barcelona, Spain; espadaler@ab-biotics.com

**Keywords:** probiotic bacteria, monacolin K, *Lactoplantibacillus plantarum*, LDL-cholesterol, bile-salt hydrolases, statins

## Abstract

Background: Dietary supplements have been proposed to help manage blood cholesterol, including red yeast rice (RYR) extracts, plant sterols and stanols, beta-glucans, and some probiotics. This study was conducted to evaluate the efficacy of RYR (containing 10 mg of monacolin K) combined with 10^9^ CFU of three *Lactoplantibacillus plantarum* strains (CECT7527, CECT7528, and CECT7529). Methods: A 12-week randomized, double-blinded, placebo-controlled clinical trial was conducted. In total, 39 adult patients were enrolled, having total cholesterol (TC) ≥200 mg/dL, and being statin-naïve or having recently stopped statin treatment because of intolerance. Active product or placebo were taken once daily, and subjects were evaluated at baseline, 6, and 12 weeks. Results: Study groups were comparable at baseline, except for history of recent hypercholesterolemia treatment (81% in active vs. 22% in placebo). Changes in LDL cholesterol and TC became significant compared to placebo (mean difference between groups and standard error of the mean = 23.6 ± 1.5 mg/dL, *p* = 0.023 and 31.4 ± 1.9 mg/dL, *p* = 0.011, respectively) upon adjusting for the baseline imbalance in hypercholesterolemia treatment. No adverse effects were noted during the study. Conclusion: This combination of 10 mg of monacolin K and *L. plantarum* strains was well tolerated and achieved a statistically significant greater reduction in LDL-C and TC in the intervention group compared to the placebo, once adjusting for recent history of hypercholesterolemia treatment.

## 1. Introduction

Cardiovascular diseases (CVDs) are the number one cause of death globally, representing 31% of all global deaths [1]. The retention of low-density lipoprotein cholesterol (LDL-C) and similar cholesterol-rich lipoproteins containing apolipoprotein B (ApoB) within the arterial wall is a key initiating event in atherogenesis [2]. Moreover, compelling evidence shows that elevated low-density lipoprotein cholesterol (LDL-C) level is a major modifiable risk factor for CVD [3], thus making LDL-C a major target for risk reduction [4,5].

LDL-C levels are determined by multiple dietary, genetic, and environmental factors [6], and may be corrected through an adequate lifestyle (diet and physical exercise) and, if necessary, an appropriate drug treatment. Statins (3-hydroxy-3-methylglutaryl-CoA reductase inhibitors) are the mainstay of pharmacological cholesterol-reduction therapy [4,5]. Statins are the mainstay of atherosclerosis treatment with a reduction in cardiovascular risk, CVD, and mortality, with a risk–benefit profile that appears to differ according to statin type, age, and gender [7]. In this line, concerns have been raised regarding statin-related adverse effects, such as statin-associated muscle symptoms (SAMSs), reported in 5% to 20% of patients [5,8] with the diagnosis of this disorder largely based on the presence of subjective symptoms reported by the patient [9]; increased relative and absolute risk of renal and liver dysfunction [10]; dose-dependent worsening of glycemic control in diabetic patients [11], among other things. Thus, patients may refuse to use statins despite their elevated LDL-C levels [12,13].

Growing attention has been devoted to the correction of increased LDL-C levels through the use of dietary supplements, either because some patients have milder forms of hypercholesterolemia or as an alternative to statins in patients who may have experienced or are worried about side effects [14]. The most studied nutraceuticals include monacolin K (a structural analogue of lovastatin) found in red yeast rice (RYR), plant sterols and stanols, and beta-glucans [14,15]. The gut microbiota has also been implicated in the regulation of host cholesterol homeostasis [16,17]. Accordingly, probiotics, defined as “live microorganisms which when administered in adequate amounts confer a health benefit on the host” [18], have also been studied for their effect on lipid metabolism and cholesterol-lowering effects. In this regard, a meta-analysis showed some of them may have a significant effect on blood cholesterol, mostly pertaining to species *Lactoplantibacillus plantarum* and *Limosilactobacillus reuteri* [19], formerly known as *Lactobacillus plantarum* and *Lactobacillus reuteri* [20].

Nutraceutical combinations are increasingly used in clinical practice, and thus deserve proper evaluation in clinical trials [21,22]. In this pilot study, we sought to evaluate the effect of a novel nutraceutical (combining 10^9^ colony-forming units (CFUs) of three *L. plantarum* strains (CECT7527, CECT7528, and CECT7529) and RYR extract containing 10 mg of monacolin K) against placebo on LDL-C and other blood lipid parameters of hypercholesterolemic subjects, being statin-naïve or having recently stopped statin treatment because of reported intolerance.

## 2. Materials and Methods

### 2.1. Study Design and Ethics

This randomized, double-blinded, placebo-controlled clinical trial was conducted at the Merida University Center of University of Extremadura. The study adhered to the tenets of the Declaration of Helsinki and was approved by the Internal Review Board (IRB) of the University of Extremadura (protocol ID number: 35/2014—approved on 6 May 2014). The protocol was retrospectively registered on 21 December 2020 at ClinicalTrials.gov (NCT04677335). All participants gave written informed consent before enrolment in the trial. Patients’ data were stored dissociated from the personal identification of the subjects so that the study data were identified with a numerical code, which ensured that the information handled did not contain personal data. The correspondence between codes and subjects’ identification data was kept in a separate place and only accessible to the coordinating investigator and/or investigators responsible for the center or to the corresponding authorities of the research ethics committees with medicines (RECm).

### 2.2. Study Procedures

The study was advertised in primary care centers, and patients with raised total cholesterol (TC) levels who were interested in the study were referred to the study site (Merida University Center) by their treating physicians. The study was scheduled across 4 visits: Visit 0 (week −1, prescreening), visit 1 (week 0, baseline), visit 2 (week 6), and visit 3 (week 12). A fasting blood sample was taken at the prescreening visit to confirm blood lipids data. In the baseline visit, we explained the objectives and procedures of the study, and patients who met all selection criteria and provided written informed consent were included in the study. We also collected demographic data, clinical history, waist perimeter, body mass index (BMI), body weight, and percent body fat in this baseline visit, and patients were randomized to active treatment or placebo. All patients were given the same dietary recommendations (from the Spanish Endocrinology and Nutrition Society), verbally and in writing. They were informed about the nutritional and dietary recommendations for the prevention of atherosclerosis and were provided with information on the appropriate diet for patients with dyslipidemia or hypercholesterolemia with foods allowed, to be limited or discouraged. The average nutritional value of energy per day was estimated at 2000 kcal. In visits 2 and 3, we collected follow-up data on blood lipids (from a fasting blood sample), and safety and treatment compliance were checked. Body mass index (BMI), body weight, percent body, and patient satisfaction were also recorded on visit 3 (end of the intervention).

Patients were randomized 1:1 using a computer-generated randomization list stratified by age, sex, and BMI to receive either: (i) active capsules; or (ii) placebo capsules. One cut-off point for each variable was established (age cut-off: 56; BMI cut-off: 26) for stratification purposes. The randomization list was generated by a statistics professor at the University of Extremadura not otherwise involved in the study. Active and placebo capsules were indistinguishable and were delivered in identically looking anonymous blisters and packaging. Both patients and caregivers were blinded to treatment allocation during the whole duration of the study. Patients were instructed to take one capsule a day, either after breakfast or after dinner, for 12 weeks. Subjects were informed that they should immediately report any changes in their treatment, clinical, or laboratory status during the course of the trial (all subjects were provided with a direct telephone access number to the principal investigator).

### 2.3. Study Products

Active product was composed of 10^9^ CFU of *L. plantarum* strains CECT7527 (KABP011™), CECT7528 (KABP012™), and CECT7529 (KABP013™) in a 1:1:1 proportion plus RYR extract certified to contain 10 mg of monacolin K, in a maltodextrin carrier. Conversely, placebo capsules contained maltodextrin alone. Both active and placebo were manufactured by ALIFARM SL (Barcelona, Spain) under Good Manufacturing Practices (GMPs), and tested for total live lactic acid bacteria, as well as tested for the absence of the following contaminants: *Escherichia coli*, *Salmonella*, *Staphylococcus aureus*, yeasts and molds, and heavy metals. Moreover, the red yeast rice (*Monascus purpureus*) extract was certified for absence of citrinin (less than 25 ppb) following EU regulations EC1881/2006 and EC1901/2019. Both the active treatment and the placebo were identical, the only difference being the barcodes on the outer packaging.

### 2.4. Study Population and Sample Size

Patients were included according to the following criteria: men and women, aged between 18 and 70 years who provided written informed consent, had total cholesterol (TC) ≥ 200 mg/dL, and were statin-naïve or had recently stopped statin treatment because of statin intolerance. A washout of two weeks was required for those patients who had just stopped their statin therapy [23–25].

Exclusion criteria included history of cardiovascular events or alcohol abuse, presence of diabetes, chronic advanced kidney disease, thyroid disorders, hepatic disorders, familial hypercholesterolemia, immunosuppression, body mass index (BMI) ≤ 18.5 or ≥40 kg/m^2^, use of antibiotics within 4 weeks of study initiation, current use of other probiotics, lipid-lowering medications, corticoids, beta-blockers or calcium channel blockers, thiazide diuretics, estrogen replacement therapy, pregnant or lactating women, or patients with other severe disease that could interfere with the results of the study. Although the use of angiotensin-converting enzyme inhibitors (ACE inhibitors) or angiotensin receptor blockers (ARBs) do not appear to be entirely lipid-neutral agents [26], none of the study participants were taking these drugs. Patients had to agree to maintain their usual physical activity throughout the study.

Calculations of minimally detectable effect sizes, as well as of sample sizes for the present study, were performed with the G*Power software (version 3.1.9, Universität Düsseldorf, Düsseldorf, Germany) [27]. Probiotic properties are thought to be strain specific, and prior data on the combined effect of this particular probiotic formula with RYR were not available to undertake a sample size calculation. Therefore, we designed this pilot study with a size of 40 patients. Assuming a drop-out rate of 10%, this sample size would allow the detection of a moderate-to-large effect size (anticipated Cohen’s *f* = 0.3 to 0.4) with alpha = 0.05 and beta = 0.80 in a repeated measures general linear model (GLM) with 2 groups and 3 timepoints, depending on the correlation between timepoints [28]. Several simulations were performed for a within-between design. The a priori sample size was calculated assuming equal distribution and correlations as low as 0.1 and non-sphericity epsilon of 0.8, or correlation of 0.2 and non-sphericity of 0.7, both yielding *n* = 40 for an effect size of 0.3.

### 2.5. Study Outcomes

The aim of this study was to evaluate the efficacy and safety of the nutraceutical combining monacolin K and *L. plantarum* probiotic strains on blood lipids, compared to placebo. The primary outcome of the study was the difference in evolution among groups of LDL-C across the study. Secondary efficacy outcomes were the evolution across the study of TC (main secondary outcome), high-density lipoprotein cholesterol (HDL-C), triglycerides (TG), BMI, body weight, and percent body fat. The latter three parameters were determined with a MC780 Body Analyzer (Tanita EU, Amsterdam, The Netherlands). Secondary outcomes also included: (i) adverse effects throughout the study, their occurrence and relatedness documented according to the Spanish Medicine and Medical Device Agency (AEMPS) pharmacovigilance system; and (ii) treatment satisfaction at the end of the study (visit 3), rated with a Likert-type scale ranging 0 (very dissatisfied) to 4 (very satisfied).

### 2.6. Statistical Analyses

The IBM^®^ SPSS^®^ Statistics^®^ (version 20.0) statistics program was used for statistical analyses. Data normality was checked with the Shapiro–Wilk test. Follow-up blood lipids data as well as BMI and percent body fat were analyzed by means of a repeated measures general linear model (GLM) with a within-between design. Because a stark imbalance was observed in baseline data regarding history of recent hypercholesterolemia treatment, GLMs were performed both unadjusted and adjusted for the said factor, as recommended by the European Medicines Agency (EMA) guideline [29]. Additionally, within-group changes in all variables were further evaluated by unadjusted repeated measures GLM performed separately for each treatment arm. GLM residuals were checked for normality and homoscedasticity using P-P plots and scatterplots, respectively, while absence of multicollinearity was checked by variance inflation factors (VIFs). For other analyses, the Fisher test was used for categorical data, student T-test was used for continuous parametric data, and Mann–Whitney test was used for continuous non-parametric data as well as ordinal data (i.e., alcohol consumption, product satisfaction, and side effects). The threshold for significance was set at two-sided alpha = 0.05 for all analyses.

## 3. Results

### 3.1. Study Sample

**Figure 1:**
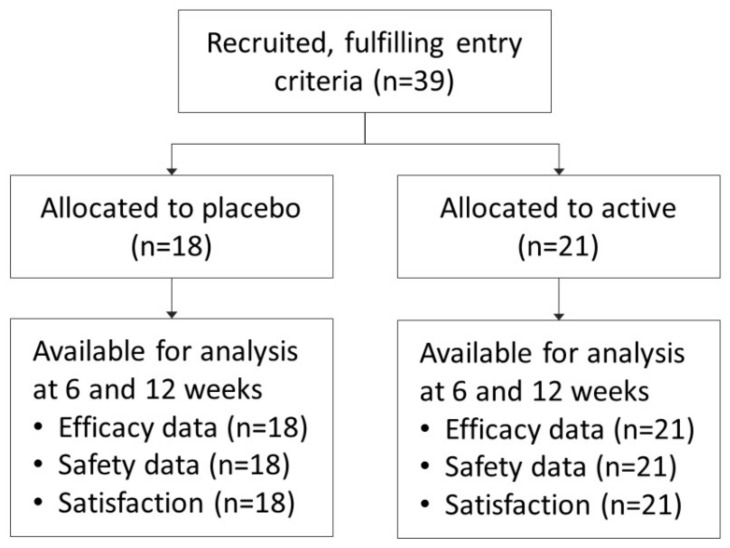
CONSORT flowchart of recruited patients.

A total of 39 subjects were included in the study, 21 in the active group and 18 in the placebo one. The study was conducted from October 2014 to September 2017. None of them dropped out from the study, and full data was available for all patients at all follow-up visits (Figure 1). Most participants were men (*n* = 25, 64%), and age ranged 32 to 69 years (median 55 years).

Study groups were comparable regarding total cholesterol, LDL-C, and HDL-C at baseline, as well as age, sex, BMI, body weight, waist circumference, smoking status, and alcohol consumption (Table 1). A trend towards a difference at baseline was noted for triglycerides and percent body fat. However, a striking difference was found regarding history of recent hypercholesterolemia treatment: 17 out of 21 subjects in the active group (81%) had been recently treated for hypercholesterolemia before entering the study, opposed to only 4 out of 18 subjects in the placebo group (22%). Baseline LDL-C levels were higher in the group with previous hypercholesterolemia treatment than in those without (163.5 ± 20.5 vs. 147.1 ± 21.7 mg/dL, *p* = 0.020). Said treatments consisted of dietary interventions and/or statin therapy of low (lovastatin 20 mg) or moderate intensity (atorvastatin 20 mg) but had stopped before entering the study as per entry criteria.

### 3.2. Efficacy

The efficacy sample comprised all 39 randomized patients. In unadjusted analysis, none of these changes were significant when comparing among groups.

**Figure 2:**
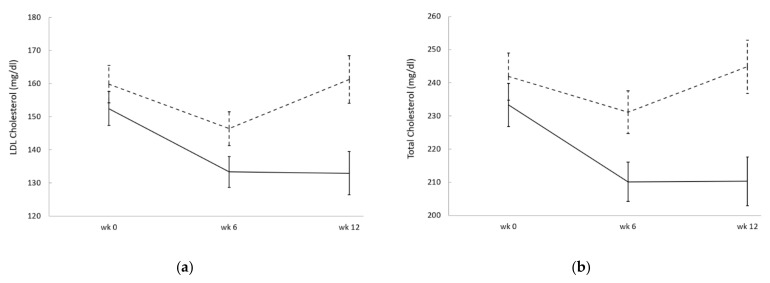
Mean blood levels of LDL-C (**a**) and TC (**b**) in the placebo (dashed line) and active (continuous line) groups, adjusted for history of recent hypercholesterolemia treatment. Error bars indicate standard errors of the adjusted means. In repeated measures general linear model analysis (GLM), the effect of treatment was statistically significant both in LDL-C (*p* = 0.023) and TC (*p* = 0.011), as well as the effect of history of recent hypercholesterolemia treatment (*p* < 0.001 for both).

However, upon adjusting for the baseline imbalance in the history of recent hypercholesterolemia treatment, the overall reductions in LDL-C and TC in the active group became significantly larger than placebo (*p* = 0.023 and *p* = 0.011, respectively; Figure 2). The effect of history of recent hypercholesterolemia treatment was highly significant in the GLMs of both outcomes (*p* < 0.001), and the achieved statistical power was *β* = 64.5 and *β* = 74.2 for LDL-C and TC, respectively. Accordingly, sample size calculations with alpha = 0.05 at 80% power indicate 27 and 21 subjects per group would be needed to detect such difference in LDL-C and TC, respectively, not counting drop-outs, provided study groups were well balanced. Comparing values at the end of the intervention (week 12) to baseline, the adjusted change (mean and standard error of the mean) in LDL-C was −20.7 ± 1.3 mg/dL in the active group (i.e., a 13% reduction) compared to +2.8 ± 1.5 mg/dL in the placebo, resulting in a mean difference of 23.6 ± 1.5 mg/dL between groups. Similarly, the adjusted change of TC was −25.5 ± 1.6 mg/dL in the active group (i.e., an 11% reduction) compared to +5.9 ± 1.8 mg/dL in the placebo, resulting in a mean difference of 31.4 ± 1.9 mg/dL between groups. Conversely, no significant differences were detected between groups in HDL-C or TG upon adjusting for the baseline imbalance in the history of recent hypercholesterolemia treatment.

In the within-group repeated measures analysis (secondary outcomes), LDL-C, TC, and TG were significantly reduced in the active group across the intervention period (*p* = 0.008, *p* = 0.007, and *p* = 0.015, respectively; Table 2) but not in the placebo group, and HDL-C was significantly increased in the active group (*p* = 0.004) but not in the placebo.

Regarding BMI, body weight, and body fat percent, a significant within-group reduction was observed in the active group across the intervention period (*p* = 0.001, *p* = 0.001, and *p* = 0.003, respectively; Table 2) and a trend towards a lower body fat percent was also observed in the placebo group (*p* = 0.061). However, these effects were not statistically significant when comparing among groups, neither in the unadjusted analysis nor when adjusting for the baseline imbalance in history of recent hypercholesterolemia treatment. Sample size calculations indicate that at least 45 subjects per group would be required to detect such a difference in BMI and body weight with alpha = 0.05 at 80% power, not counting drop-outs. Furthermore, the sample size required to assess the effect on body fat would require 190 subjects per group, not counting drop-outs.

### 3.3. Safety and Product Satisfaction

The safety population comprised all 39 randomized patients. No deaths nor adverse events (AEs) were reported during the study. At the end of the study, treatment satisfaction was rated higher among subjects randomized to the active group (median rating of 4 points, range 3–4) than those randomized to the placebo (median rating of 3 points, range 2–4), this difference being statistically significant (*p* = 0.009).

## 4. Discussion

In this study, we analyzed the effect of a 12-week once-daily intervention with a combination of 10^9^ CFU of *L. plantarum* strains (namely CECT7527, CECT7528, and CECT7529) and RYR certified to contain 10 mg of monacolin K on blood cholesterol. In total, 39 patients were included, and slightly more than half of them (*n* = 21, 54%) had been recently treated for hypercholesterolemia before entering the study. Due to blinded randomization, the proportion of those allocated to the active group intervention ended up being significantly higher than the placebo (*p* < 0.001). Said hypercholesterolemia treatments had consisted of: (i) various dietary interventions, which were voluntarily switched to the allocated study intervention (active nutraceutical or placebo); or (ii) statins of low (lovastatin 20 mg) or medium (atorvastatin 20 mg) intensity, which had been discontinued prior to study initiation because of intolerance complains. This particular combination of *L. plantarum* strains was chosen because it was previously shown to lower cholesterol in a randomized, placebo-controlled clinical trial [31], as well as having high bile salt hydrolase activity [32] and good safety data [33].

Unadjusted analysis of LDL-C and TC levels across the study did not reveal significant differences between groups. However, upon accounting for the strong imbalance in history of recent hypercholesterolemia treatment, a statistically significant improvement was observed both for LDL-C and TC in the active group compared to placebo. Conversely, no significant effect was observed on HDL-C, TG, BMI, and percent of body fat.

Monacolin K is structurally equivalent to lovastatin [34], and its efficacy in reducing LDL-C has been studied in many clinical trials, with clear effects seen in meta-analyses [35,36]. However, several concerns have been raised about the lack of standardization and safety of RYR, including the possible presence of the nephrotoxic mycotoxin citrinin [37]. However, a recent meta-analysis of 53 randomized clinical trials totaling 4437 subjects treated with RYR and 4303 controls found no increased risk of SAMS compared to placebo (only RYR doses were reported but not their monacolin K content) [38]. Moreover, a significantly lower risk of non-muscular side effects was noted in subjects receiving RYR. Nevertheless, care was taken in our study to utilize an RYR extract certified for 10 mg of monacolin K and for being devoid of citrinin according to EU regulations EC1881/2006 and EC1901/2019.

Various mechanisms have been proposed for bacteria with high bile salt hydrolase (BSH) activity (such as *L. plantarum* strains CECT7527, CECT7528, and CECT7529) in lowering blood cholesterol. These include: (i) reducing apical sodium-dependent bile acid transporter (ASBT) transporter-mediated intestinal reabsorption of bile salts due to its lower affinity for deconjugated bile salts [39]; (ii) increased expression of host genes involved in the cholesterol efflux system (*Abcg5/8*), lipid metabolism (*Pparγ*, *Angptl4*), circadian rhythm (*Dbp* and *Per1*/2), or intestinal homeostasis (*RegIIIγ*) [40]; and iii) changes in bile-dependent signaling on farnesoid X receptor (FXR), Takeda G-protein receptor 5 (TGR5), or vitamin D receptor (VDR) [41].

The clinical effect of the specific combination of *L. plantarum* strains used in this study was previously assessed in a randomized, double-blinded trial in a 12-week intervention with probiotic alone or placebo [31]. The said study enrolled 60 patients and found a similar reduction in LDL-C and TC compared to baseline (14% and 13%, respectively), which are slightly reduced when accounting for the placebo effect (to 8% and 9%, respectively). LDL-C and TC reductions from baseline averaged 13% and 11% in the present study, which are increased to 15% and 13% when accounting for placebo. These results seem logical, since the present study combines both the probiotic and monacolin K. Conversely, the former study observed a significant reduction in TG (16% vs. baseline and 14% vs. placebo), as opposed to the present study. However, baseline TG levels averaged 180 mg/dL in the former study, compared to 143 mg/dL in the present one. More recently, an observational study was conducted involving more than 340 patients taking the same *L. plantarum* strains, alone or in combination to pre-existing statins at a stable dose of moderate or high intensity [42]. Subjects initiating statin therapy were not allowed in the study. In those subjects taking probiotic alone, a larger reduction from baseline was observed in LDL-C (23%) compared to the present study. However, the lack of randomization and treatment concealment makes direct comparisons difficult. Additionally, a significant reduction in TG was also observed (22% vs. baseline), but baseline TG levels were much higher in said study (averaging 340 mg/dL), compared to the present study. Of note, subjects who added the probiotic treatment to already ongoing statin therapy obtained even larger reductions in LDL-C, averaging 29%, and no side effects were noted compared to those taking probiotic alone. Taken together, the results of these studies suggest these probiotic strains may have a beneficial effect on TG in subjects with raised levels at baseline (the higher the baseline, the larger the effect) but not in subjects with normal (i.e., <150 mg/dL) or borderline TG levels. This observation should be verified in future clinical studies. Additionally, since monacolin K is known to be structurally equivalent to lovastatin [34], the former observational study [42] and the present randomized study support the safety of combining this particular probiotic formula to statin-type therapy.

Few other studies have assessed the effect of probiotics combined with monacolin K on blood cholesterol. Kullisaar and colleagues studied a different probiotic strain (*Lactobacillus fermentum* ME-3) at a higher dose (6 × 10^9^ CFU) together with RYR containing 10 mg of monacolin K, 30 mg of coenzyme Q10, 30 mg of L-cysteine, and vitamins B1, B6, B9, B12, and E [43]. Their study reported a significant decrease of TC and LDL-C lipids at 4 weeks; however, the study was unblinded and lacked a placebo group. Additionally, product intake was twice a day, while our intervention was once daily. More recently, Ruscica and colleagues studied a different probiotic strain (*Bifidobacterium longum* BB536) at an equivalent dose to this study (10^9^ CFU), together with RYR containing 10 mg of monacolin K, 16 mg of vitamin B3 (niacin), and 20 mg of coenzyme Q10 [21]. Their study was randomized and found significant improvements in LDL-C and TC, which were seemingly larger than in our study. However, their study population also had higher LDL-C and TC at baseline than ours and excluded subjects with BMI equal or higher than 30 kg/m^2^, while 20% of our of study sample (*n* = 8) had a BMI in the 30 to 38 kg/m^2^ range. Additionally, no mention was made of recent treatments of hypercholesterolemia. As a result, these differences make any direct comparison difficult. Recent treatment of hypercholesterolemia could in fact be considered as a prognostic factor, as those who needed cholesterol-lowering treatment might have had higher baseline cholesterol or experienced the rebound effect of statins. This fact was taken into account in our study. However, it is worth noting that, as in our study, Ruscica and colleagues observed that both HDL-C and TG were unaffected by the intervention compared to placebo [21].

This study presents some limitations inherent to the fact of it being a pilot study. First, the small sample size resulted in a marked imbalance in history of recent hypercholesterolemia treatment between study groups. However, statistical adjustment was performed to account for this factor, and checkups were performed to ensure the statistical soundness of the resulting general linear model. Second, the small sample size precluded subgroup analysis being performed depending on the type of hypercholesterolemia treatment used prior to entering the study (i.e., nutritional intervention, low-potency statin, or moderate-potency statin). Third, the effect on inflammation markers (hsCRP, IL-6) or additional markers related to blood cholesterol (PCSK9, Apo-B, oxidized LDL-C) was not evaluated. Fourth, the protocol was retrospectively registered at ClinicalTrials.gov.

Although there were statistically significant differences in product satisfaction in the active group versus the placebo group, we cannot explain why the differences in scores were due to this, as the masking was not broken at any time (patients were unaware of the results of their tests and their data were not incorporated into their clinical records, as they were performed independently). One of the hypotheses could be due to the greater body weight loss and fat mass percentage in the active group compared to the placebo [44,45].

However, the study shows promising results on LDL-C and TC with once daily treatment with this particular probiotic and monacolin K combination, especially for patients who complain of statin intolerance. To our knowledge, this is the first study with a probiotic-containing nutraceutical to include this type of patients in the study population. The fact that 10 mg of monacolin K (structurally analogous to lovastatin) combined with this probiotic was tolerable while achieving a significant reduction in both LDL-C and TC in patients who had recently stopped taking higher doses of pharmaceutical-grade statins (20 mg of lovastatin or same dose of atorvastatin) suggests this particular nutraceutical combination can be useful for said patients. In our view, these results warrant further randomized studies of a larger size. Preferably, such studies should enroll patients in a stratified manner according to the existence and intensity of prior hypercholesterolemia treatment. Additiionally, it would be desirable to enroll subjects both with normal and markedly raised TG levels at baseline in a stratified manner. Finally, small changes could be seen in BMI, body weight and body fat during the 3-month intervention of this pilot study, which achieved within-group significance in the active group but not placebo. Sample size calculations indicate that at least 90–100 subjects would be necessary to confirm with 80% statistical power whether the particular combination of *L. plantarum* strains and monacolin K used in this study helps reduce body weight and BMI against placebo. In the placebo group, average LDL-C and TC levels were seemingly reduced at 6 weeks but not at 12 weeks. Dietary recommendations and exercise are the first line of treatment for people with elevated cholesterol values; however, these methods can only achieve a modest improvement and adherence fades away with time, a pattern that may explain what happened in our placebo group [24,46]. A longer intervention time and follow-up to assess weight-related changes and their stability over time should be proposed in future studies.

## 5. Conclusions

A 12-week pilot intervention with the combination of 10 mg of monacolin K and *L. plantarum* strains CECT7527, CECT7528, and CECT7529 was well tolerated and achieved a statistically significant reduction in LDL-C and TC against placebo. A large proportion of patients in the active group had a recent history of hypercholesterolemia treatment and had recently stopped taking statins because of intolerance complains. Therefore, this nutraceutical combination may be useful for such patients and warrants further randomized clinical trials.

## Figures and Tables

**Table 1:** Baseline characteristics of study subjects.

	Total Sample (*n* = 39)		Placebo (*n* = 18)		Active (*n* = 21)	
Age (mean and SD)	51.9	(±11.8)	48.8	(±12.2)	54.5	(±9.0)
Sex (female, %)	14	(35.9%)	5	(27.8%)	9	(42.9%)
TC (mean and SD)	237.3	(±28.9)	232.7	(±32.9)	241.1	(±25.3)
LDL-C (mean and SD)	155.9	(±22.4)	153.3	(±28.0)	158.1	(±16.6)
HDL-C (mean and SD)	55.5	(±14.1)	59.2	(±18.6)	52.4	(±7.9)
TG (mean and SD)	143	(±67.7)	122.2	(±37.4)	160.9	(±82.5)
Glycemia (mean and SD)	92.6	(±12.7)	95.8	(±10.0)	89.8	(±14.3)
Hemoglobin (mean and SD)	15.6	(±1.1)	15.8	(±1.1)	15.5	(±1.2)
Waist perimeter (mean and SD)	96.9	(±11.1)	95.9	(±13.4)	97.7	(±8.9)
Body Mass Index (mean and SD)	27.1	(±4.1)	26.6	(±4.5)	27.5	(±3.8)
Body Fat (%) (mean and SD)	27.8	(±6.2)	26.0	(±4.4)	29.3	(±7.2)
Smoking habit (yes, %)	14	(35.9%)	5	(27.8%)	9	(42.9%)
Alcohol consumption ^1^ (yes, %)	25	(64.1%)	11	(61.1%)	14	(66.7%)
Antihypertensive treatment (yes, %)	1	(2.6%)	1	(5.6%)	0	(0.0%)
Recent hypercholesterolemia treatment (yes, %)	21	(53.8%)	4	(22.2%)	17	(81.0%)
Family history of hypercholesterolemia (yes, %)	13	(33.3%)	6	(66.7%)	7	(66.7%)

Data expressed as mean (± standard deviation) and frequencies (percentages). Abbreviations: SD: Standard Deviation; TC: Total Cholesterol; LDL-C: Low-Density Lipoprotein Cholesterol; HDL-C: High-Density Lipoprotein Cholesterol; TG: Triglycerides; ^1^ A *“standard drinking unit”* is equal to 10 g of pure alcohol [30].

**Table 2:** Change from the baseline (week 0) of study outcomes, with repeated measures within-group *p*-values.

	Placebo (*n* = 18)			Active (*n* = 21)		
	Mean	SEM	*p*-Value	Mean	SEM	*p*-Value
TC (mg/dL), week 6	−13.4	6.4	0.766	−20.9	5.7	0.007
TC (mg/dL), week 12	−2.8	9.4		−18.0	6.0	
LDL-C (mg/dL), week 6	−17.1	5.8	0.571	−16.0	5.0	0.008
LDL-C (mg/dL), week 12	−3.6	6.2		−15.3	5.2	
HDL-C (mg/dL), week 6	−1.2	1.3	0.847	0.1	1.4	0.004
HDL-C (mg/dL), week 12	0.4	2.0		3.1	1.0	
TG (mg/dL), week 6	3.3	10.1	0.370	−32.4	15.1	0.015
TG (mg/dL), week 12	10.7	11.6		−30.3	11.4	
Body weight (kg), week 12	−0.4	0.4	0.249	−1.4	0.4	<0.001
BMI (kg/m^2^), week 12	−0.2	0.1	0.215	−0.5	0.1	0.001
Body Fat (%), week 12	−0.8	0.4	0.061	−1.3	0.4	0.003

Statistically significant *p*-Values are indicated in bold. Abbreviations: SEM: Standard Error of the Mean; TC: Total Cholesterol; LDL-C: Low-Density Lipoprotein Cholesterol; HDL-C: High-Density Lipoprotein Cholesterol; TG: Triglycerides; BMI: Body Mass Index.

## Data Availability

The data are not publicly available due to the fact that they may contain sensitive patient data.
